# Estimating the probabilities of making a smoking quit attempt in Italy: stall in smoking cessation levels, 1986-2009

**DOI:** 10.1186/1471-2458-12-183

**Published:** 2012-03-12

**Authors:** Giulia Carreras, Silvano Gallus, Laura Iannucci, Giuseppe Gorini

**Affiliations:** 1 Environmental and Occupational Epidemiology Unit, ISPO Cancer Prevention and Research Institute, Ponte Nuovo - via delle Oblate, 1-50141 Florence, Italy; 2 Department of Epidemiology, Mario Negri Institute for Pharmacological Research, Via La Masa, 19-20156 Milan, Italy; 3 Italian National Institute of Statistics (ISTAT), Via Ravà, 150-00142 Rome, Italy

## Abstract

**Background:**

No data on annual smoking cessation probability (i.e., the probability of successfully quit in a given year) are available for Italy at a population level. Mathematical models typically used to estimate smoking cessation probabilities do not account for smoking relapse. In this paper, we developed a mathematical model to estimate annual quitting probabilities, taking into account smoking relapse and time since cessation.

**Methods:**

We developed a dynamic model describing the evolution of current, former, and never smokers. We estimated probabilities of smoking cessation by fitting the model with observed smoking prevalence in Italy, 1986-2009.

**Results:**

Annual cessation probabilities were higher than 5% only in elderly persons and in women aged < 30 years, while in adults aged 30-49 and 50-59 cessations were about 2% and 3-5%, respectively. Most of quit probabilities stalled from 1986 to 2009.

**Conclusions:**

Over the last 20 years, cessation probabilities among Italian smokers, particularly for those aged 30-59 years, have been very low and stalled. Quitting in Italy is considered as a practicable strategy only by women in the age of pregnancy and by elderly persons, when it’s likely that symptoms of tobacco-related diseases have already appeared. In order to increase cessation probabilities, smoking cessation treatment policies (introducing total reimbursement of cessation treatments, with a further development of quitlines and smoking cessation services) should be empowered and a country-wide mass media campaign targeting smokers aged 30-59 years and focusing on promotion of quitting should be implemented.

## Background

Smoking is a major risk factor for many tumours and other chronic diseases, and responsible for the reduction of length and quality of life [1]. In Italy, women smoking prevalence declined from 19.2% in 1986 to 17.0% in 1993, and afterwards stalled at that level, while men smoking prevalence declined from 41.6% in 1986 to 29.5% in 2009, with an average annual drop of 1.2% [2]. The reduction in smoking prevalence derived from the joint effect of changes in smoking initiation and cessation. Data from multipurpose surveys carried out by the Italian Institute of Statistics (ISTAT), showed a 15% decrease in smoking initiation from 8.6% in 1994 to 7.3% in 2005, for girls aged 18-25 years, and a 28% reduction, from 16.3% to 11.7%, for boys [2].

Cessation can be estimated using surveys on smoking habits [3-6]. However, repeated and comparable surveys on smoking habits over several decades are not always available. Alternatively, cessation probability could be estimated using data from representative cross-sectional surveys through the use of mathematical models [7-9] that are essentially based on a set of equations describing the demographic evolution by smoking habits, depending on assumptions on the development of smoking epidemic and on related parameters.

The knowledge of historical cessation trends (in addition to data on initiation and prevalence) is necessary to characterize the dynamics of the tobacco epidemic [3]. Previous models [7-9] estimated cessation probabilities through an optimization procedure by selecting the parameters that best reproduced the country-specific observed prevalence figures. However, these models did not take into account the smoking relapse, thus underestimating the real cessation probabilities. The success of quit attempts, and therefore the relapse of smoking, is an important aspect of smoking cessation.

In this paper, we develop a mathematical model to describe the Italian observed smoking prevalence in the years 1986-2009. Our aim is to estimate the annual cessation probabilities for Italian smokers by taking into account smoking relapse and time since smoking cessation.

## Methods

### Model

**Figure 1:**
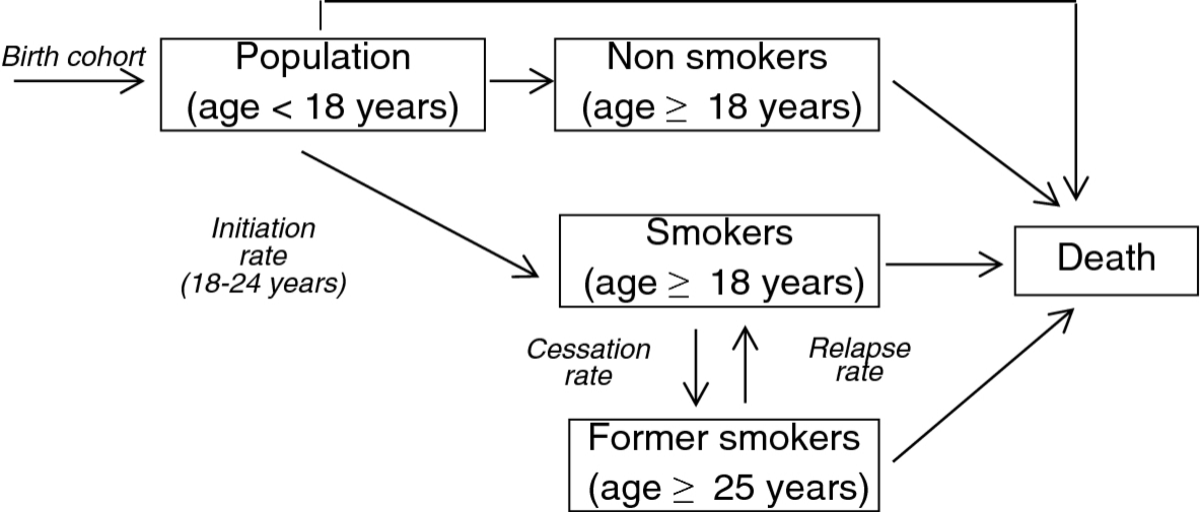
**Model**. Scheme of the model used to compute the prevalence of smoking.

We implemented a model of Italian smoking habits (Figure 1), extending the work in [9]. In defining the model, the population was stratified into three mutually exclusive groups: current, never, and former smokers. The latter group differs from the model in [9] for time since smoking cessation. Current smokers were defined as those who smoke ≥ 1 cigarette/day (i.e., both heavy and light smokers), and former-smokers were defined as those that currently do not smoke but have smoked in the past [2].

We simulated the model in order to describe the dynamics of the population over time, starting with the number of current, never, and former smokers observed in year 1986. The population’s evolution is described through a demographic model that reports the annual changes of the population numbers by gender and age, taking into account births and deaths. The number of newborns in each year was assumed to be the number of children aged 0 observed in that calendar year. The proposed model is based on a set of time-continuous differential equations, which describe the 1-year changes in the prevalence of never, current, and former smokers, specified by gender and age. The model was run for time period 1986-2009, and for each year the model produced age and sex-specific prevalences for current and former smokers.

### Data

Demographic data such as all cause mortality rates and initial baseline population numbers were available from the Italian Institute of Statistics (ISTAT). Smoking prevalence and initiation rate data by gender and age were available from the ISTAT Multipurpose Surveys “Health conditions and access to health services” and “Aspects of daily living” [2]. These surveys were carried out almost every year from 1986 to 2009 on representative samples of the Italian population. Smoking prevalence and initiation rate figures were linearly interpolated for the missing years. Figures on the proportion of former smokers by time since quitting were obtained from 10 surveys carried out annually in 2001-2010 by DOXA, the Italian branch of the Gallup International Association, on representative samples of the Italian population aged ≥ 15 years [10]. Five categories of time since cessation were considered: 1-2 years, 3-5 years, 6-10 years, 11-15 years, and > 15 years. Mortality rates for current and never smokers were derived from the Cancer Prevention Study II [11].

### Assumptions

Since data on smoking initiation rates show a fall after 24 years of age, we assumed that smoking initiation in the model occurs between 18 and 24 years. Cessation was tracked from age 25, since the relative risks of death for smoking-related diseases are not discernable for those who quit smoking before that age [11].

Relapse rates were assumed to follow a negative-exponential curve depending on the time since quitting [12,13]:

(1) $$\lambda_{relapse}\left(t\right)=\alpha \beta \text{exp}\left(-\beta t\right)$$

**Figure 2:**
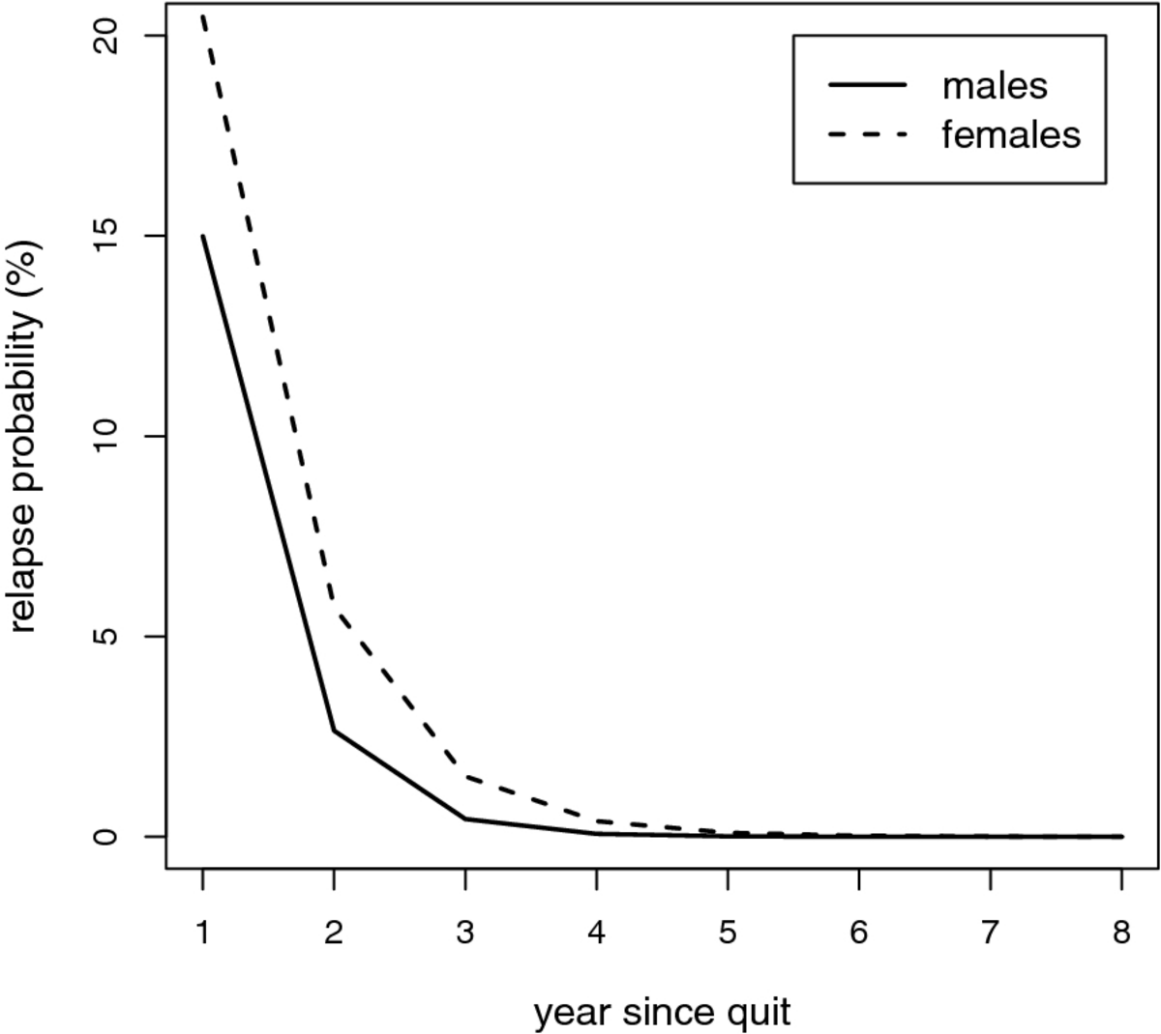
**Relapse probability**. Annual probabilities of relapse (conditional on no relapse until that time) for females and males.

The parameter *β *governs how fast the relapse rates decline with time since smoking cessation, and the parameter *α *governs the lifetime probability of no relapse. Under this assumption, the proportional decrease of relapse rates is constant over time. Thus, the relapse rates are highest shortly after cessation, and diminish in the long run. Since data on relapse were not available for Italy, we used the *α *and *β *parameters estimated from a series of cross-sectional population surveys on smoking behaviour for the Netherlands and we converted the relapse rates in annual probabilities of relapse (conditional on no relapse until that time) [12] (Figure 2).

**Figure 3:**
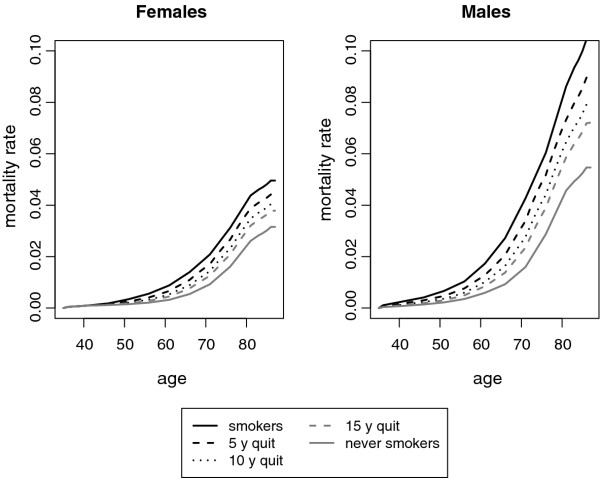
**Death rates**. Death rates for current smokers, never smokers, and former smokers by time since quit smoking and gender.

We assumed that relative risks for former smokers decrease according to a negative-exponential curve with a convergence rate decreasing with age [12]. Relative risks of former smokers are therefore allowed to gradually decrease over time since cessation being similar to those of current smokers immediately after cessation, and becoming similar to those of never smokers in the long run. As for relapse rates, we parameterized the negative-exponential curve according to estimates from a series of cross-sectional population surveys on smoking behaviour from the Netherlands [12]. We then converted relative risks into death rates (Figure 3).

### Estimation procedure

We estimated sex and age-specific annual cessation probabilities for each year in three time periods (1986-1992, 1993-1999, 2000-2009) by selecting the set of parameters that best reproduced the age-specific observed smoking prevalence [7-9]. The bounded weighted least squares method was used as optimization procedure [7-9]. The inverse variances of the observed age-specific prevalence estimates were used as weights to take into account the age-composition of the population [7-9], and the parameters were constrained in the interval [0,1] [9].

Intuitively, since the model output are smoking prevalences and the cessation probabilities are necessary input parameters, the model was run several times with a set of plausible values of cessation as input parameters. The values of cessation that let the model to reproduce the prevalences closest to the observed ones were then selected.

We used a probabilistic sensitivity analysis (PSA) approach [14] to account for uncertainty of the smoking prevalence data: prevalence of current, former, and never smokers were assumed to jointly follow a probabilistic distribution function. PSA was based on a simulation approach: for each iteration, we randomly assigned a value for the prevalence estimates of current, former, and never smokers within its probabilistic distribution. We then applied the optimization procedure and we estimated a set of cessation probabilities for each iteration. The resulting variability in the cessation probabilities was then suitably summarized. We selected the Dirichlet distribution, generally used for several mutually exclusive events, as the joint distribution function for prevalence [8,9,14]. We parameterized the distribution by a vector of positive real numbers that coded information on event rates: the observed probabilities of being current, former or never smokers were assumed as parameters. Note that Dirichlet distribution parameters are related to the mean and the variance of the event rates. Likewise, multiplying such parameters by a scale factor, a Dirichlet distribution with the same expected value and a different variability could be obtained. In this application, we tuned the observed prevalence by a scale factor to assure that the resulting cessation probability estimates were normally distributed, in order to easily estimate the mean and build confidence intervals for the cessation probabilities. Normality was tested with the Shapiro-Wilk test [15].

## Results

Estimates of annual cessation probabilities and corresponding confidence intervals are reported in Table 1. The mean point estimates produced a good fit with an R^2 ^of 0.972 for women and 0.977 for men. Annual cessation probabilities in both genders were higher in the oldest age group: over the last period (2000-2009), probabilities in women and men aged ≥ 60 years were 8.8% and 8.5%, respectively (Table 1). Annual cessation probabilities for women aged < 30 years were similar to those estimated for elderly women, around 8-10%, while in young men cessation was around 5%. Cessation probabilities in both women and men aged 30-49 years were very low, around 2%, while in women and men aged 50-59 years were around 3-5%.

**Table 1:** Annual cessation probabilities estimates and corresponding 95% confidence interval by sex and time period. Italy 1986-2009

		Women			Men	
**age group**			**Time period**			
**(years)**	**1986-1992**	**1993-1999**	**2000-2009**	**1986-1992**	**1993-1999**	**2000-2009**

< 30	7.5 (6.4, 8.6)	10.1 (9.6, 10.6)	8.9 (7.9, 9.9)	6.2 (5.5, 6.9)	4.6 (3.7, 5.4)	4.8 (4.2, 5.3)
30-39	2.6 (1.8, 3.4)	0.0 (0.0, 0.0)	3.1 (2.5, 3.7)	2.5 (2.0, 2.9)	2.4 (1.9, 3.0)	1.9 (1.5, 2.2)
40-49	3.2 (2.4, 4.0)	1.1 (0.3, 1.8)	3.4 (2.8, 4.1)	3.1 (2.6, 3.6)	2.6 (1.9, 3.2)	2.1 (1.7, 2.5)
50-59	2.3 (1.3, 3.2)	6.2 (5.2, 7.3)	3.7 (3.2, 4.3)	3.5 (3.1, 4.0)	5.0 (4.4, 5.6)	4.1 (3.7, 4.5)
≥ 60	10.2 (10.0, 10.4)	8.5 (7.7, 9.4)	8.5 (7.8, 9.2)	8.4 (8.0, 8.7)	10.2 (10.2, 10.2)	8.8 (8.4, 9.3)

Time trend of annual cessation probabilities in women aged < 30 and 50-59 years showed a slight increase, while in women aged 30-49 years stalled, and in women aged ≥ 60 years showed a slight decrease. In men aged < 50 years annual cessation probabilities slightly decreased, while in men aged ≥ 50 years cessation probabilities stalled.

For both young women and men (age < 30 years) cessation probabilities resulted the more uncertain with respect to the other age classes for the three periods. On the contrary, estimates for both women and men aged 30-39 years and ≥60 resulted the less uncertain (Table 1).

## Discussion

Estimates of annual cessation probabilities were higher than 5% only in persons older than 60 years and in women younger than 30 years, while in persons aged 30-59 years cessation estimates were around 2-5%. It’s noteworthy that most of cessation probabilities stalled from 1986 to 2009. Results may imply that quitting is considered only by women in the age of pregnancy and by elderly persons, when it’s likely that symptoms of tobacco-related diseases have already appeared. Accordingly, in Italy approximately half of former smokers endorsed present health (i.e., current health conditions) as the main reason they quit smoking [16].

The annual cessation probabilities estimated for women aged 30-39 in the time period 1993-1999 resulted very low because most of cessation was captured in the previous age class (< 30 years). In fact, younger women in 1993-1999 recorded higher cessation probabilities in comparison to the other time periods.

In our model we assumed that smoking initiation occurs between 18 and 24 years of age, even though it is frequently reported that adolescent begin to smoke before 18 years of age [17,18]. This assumption was varied by considering smoking initiation at 14 years of age, producing worst R^2 ^values (0.523 and 0.496 for women and men, respectively). Moreover, adolescent smokers are often occasional smokers (triers, puffers, experimenters), and they do not necessarily progress to regular smoking [19].

Estimates of annual cessation probabilities from our model were consistent with cessation probabilities obtained with similar models for Italy [9], for USA [7] and for Australia [8], and with survey estimates of cessation rates in the Spanish [3], Italian [4], English [5], and US population [6].

Age-standardized quit rates were estimated for Italy around 2-3% for males and 1-4% for females aged 20-44 years [4]. Cessation rates estimates for the English population over 16 years of age were between 2% and 3% for both genders in 2006 [5]. Similar analyses for Spain reported the incidence of quitting smoking of 0-5% and 1-5% for males and females aged 20-50 years old, respectively, and of 0-9% and 8-9% for males and females aged > 50 years old, respectively [3]. Cessation rates in California increased by 25% from 1980s to the 1990s, averaging 3.4% per year in the 1990s. Cessation increased for all age-groups, and by more than 40% among smokers aged 20-34 years [6]. Since in our model we estimated the annual probability of smoking cessation independently whether or not smokers successfully quitted after that year, the resulting cessation estimates were slightly higher than those reported in the other studies. The previous Italian model [9], as well as the other models and survey estimates that does not take into account time since quitting, accounted only for successful quitting (i.e., smoking abstinence for at least one year).

Empowering smoking cessation treatment policies and launching media campaigns focusing on quitting promotion among adults may increase quit attempts among Italian smokers, particularly those aged 31-59 years, whose probabilities of making a quit attempt resulted very low. Currently, the National Health System (NHS) does not reimburse smokers for pharmacotherapy or behavioral treatments [20,21], and these methods in 2011 were used by 5% of former smokers [22]. Moreover, about 40% of smokers reported having received advice to quit by their general practitioners [22,23], and each of the almost 300 NHS Smoking Cessation Services treated an average of 70-77 smokers only each year [24]. Finally, the two Italian quitlines annually received about 7-8,000 calls, thus reaching about 0.06-0.07% only of Italian smokers [25,26]. Anti-tobacco media campaigns have been implemented in Italy in 2002-2005 annually, in 2009, and 2010 [27] and their targets were young people to prevent smoking initiation. The MPOWER Report [28] indicates health expenditures of less than US$1 per person in Italy for media campaign.

### Limitations and strengths

Our model has some limitations due to simplifying assumptions or to lack of data. Migration was not taken into account because of the inadequate data on smoking prevalence among migrants. However, since the resident population is large with respect to immigrants, it is unlikely that the difference would significantly influence results although smoking prevalence among immigrants may differ from that in the resident population [10].

Despite the dependence of smoking-related mortality on many factors, as the duration of smoking and smoking intensity, the excess risk of death is modeled simply as the relative risk for current and former smokers not to increase the model complexity.

Cancer Prevention Study II mortality rates for current, and never smokers were used since Italian data were not available. However, these estimates were in agreement with those found in Italian epidemiological studies for several mortality causes [29].

In modelling relapse rates and relative risks of former smokers through the two negative-exponential curves, we used the parameters estimated from cross-sectional surveys conducted in the Netherlands since figures from Italy were not available. While relative risks for former smokers may be assumed to have similar patterns for the Netherlands and Italy, it is instead uncertain whether or not relapse in Italy follows the same pattern observed in the Netherlands.

In comparison with survey estimates of cessation rates, our model was based on the evolution of tobacco epidemic in the Italian population and provided a consistent model of how current and former smokers evolve over time. Since the knowledge of past time trends in cessation is necessary to characterize the dynamics of the tobacco epidemic, and since only few nations collect data on cessation rates, our model can be used to estimate cessation rates for those countries where this cannot be estimated from surveys on smoking habits. Moreover, the model lets to quantify the effects of smoking interventions on public health, taking into account the time since cessation of quitters. The latter is important, since many quitters relapsed and for most smoking related diseases the increased risks of former smokers only decreased gradually over time since cessation.

## Conclusions

This model estimated annual cessation probabilities among Italian smokers in 1986-2009. Results clearly showed that, over the last 20 years, cessation among Italian smokers, particularly those aged 30-59 years, was very low, around 2-5%, and stalled. In order to increase cessation rates, smoking cessation treatment policies (introducing total reimbursement of cessation treatments, with a further development of quitlines and smoking cessation services) should be empowered and a country-wide mass media campaign targeting smokers aged 30-59 years and focusing on promotion of quitting should be implemented.

## Competing interests

The authors declare that they have no competing interests.

## Authors' contributions

GC developed the mathematical model, performed the analyses and drafted the manuscript. SG participated in the acquisition of data, interpretation of results, and helped to draft the manuscript. LI participated in the acquisition of data. GG coordinated the study, participated in the interpretation of results, and drafted the manuscript. All authors read and approved the final manuscript.

## Pre-publication history

The pre-publication history for this paper can be accessed here:

http://www.biomedcentral.com/1471-2458/12/183/prepub
